# Prenatal Immune Challenge in Mice Leads to Partly Sex-Dependent Behavioral, Microglial, and Molecular Abnormalities Associated with Schizophrenia

**DOI:** 10.3389/fnmol.2018.00013

**Published:** 2018-02-08

**Authors:** Chin W. Hui, Abygaël St-Pierre, Hassan El Hajj, Yvan Remy, Sébastien S. Hébert, Giamal N. Luheshi, Lalit K. Srivastava, Marie-Ève Tremblay

**Affiliations:** ^1^Axe Neurosciences, CRCHU de Québec-Université Laval, Québec, QC, Canada; ^2^Département de Psychiatrie et Neurosciences, Université Laval, Québec, QC, Canada; ^3^Department of Psychiatry, Douglas Mental Health University Institute, McGill University, Montréal, QC, Canada; ^4^Département de Médecine Moléculaire, Université Laval, Québec, QC, Canada

**Keywords:** microglia, schizophrenia, immune challenge, behavior and cognition, gene expression, electron microscopy, morphology and physiology, ultrastructure change

## Abstract

Epidemiological studies revealed that environmental factors comprising prenatal infection are strongly linked to risk for later development of neuropsychiatric disorders such as schizophrenia. Considering strong sex differences in schizophrenia and its increased prevalence in males, we designed a methodological approach to investigate possible sex differences in pathophysiological mechanisms. Prenatal immune challenge was modeled by systemic administration of the viral mimic polyinosinic-polycytidylic acid (Poly I:C) to C57BL/6 mice at embryonic day 9.5. The consequences on behavior, gene expression, and microglia—brain immune cells that are critical for normal development—were characterized in male vs. female offspring at adulthood. The cerebral cortex, hippocampus, and cerebellum, regions where structural and functional alterations were mainly described in schizophrenia patients, were selected for cellular and molecular analyses. Confocal and electron microscopy revealed most pronounced differences in microglial distribution, arborization, cellular stress, and synaptic interactions in the hippocampus of male vs. female offspring exposed to Poly I:C. Sex differences in microglia were also measured under both steady-state and Poly I:C conditions. These microglial alterations were accompanied by behavioral impairment, affecting for instance sensorimotor gating, in males. Consistent with these results, increased expression of genes related to inflammation was measured in cerebral cortex and hippocampus of males challenged with Poly I:C. Overall, these findings suggest that schizophrenia's higher incidence in males might be associated, among other mechanisms, with an increased microglial reactivity to prenatal immune challenges, hence determining disease outcomes into adulthood.

## Introduction

Schizophrenia is a chronic and severe psychiatric disorder that is 1.4 times more frequently diagnosed in males than females, over the course of late adolescence or early adulthood (Picchioni and Murray, 2007). Male patients show an earlier age of onset, decreased social functioning, and worsened negative but reduced depressive symptoms as compared with females (Abel et al., 2010). Sex differences in antipsychotic responses were also identified, with better improvement of negative symptoms measured in men, and of affective symptoms and cognitive functions in women (Abel et al., 2010). However, sex differences in cellular and molecular mechanisms remain largely undetermined.

The disease is related to genetic (Schizophrenia Working Group of the Psychiatric Genomics Consortium, 2014; Siegert et al., 2015; Richards et al., 2016) and environmental factors (Dean and Murray, 2005; Gallagher et al., 2016), and likely triggered through a complex interplay between the two (Davis et al., 2016; Mandelli et al., 2016). Epidemiological studies have implicated prenatal infection and immune genes variations, notably of complement component 4 linked to microglial refinement of neuronal circuits, in the development of schizophrenia (Müller et al., 2015; Estes and McAllister, 2016; Hudson and Miller, 2016; Sekar et al., 2016; Srinivas et al., 2016). Animal models of maternal immune activation (mIA) using the viral mimic Poly I:C, or other immune stimuli, display neurobehavioral impairments affecting motor control, anxiety, sociability, memory, and sensorimotor gating that are reminiscent of schizophrenia symptoms (Jones et al., 2011; Meyer, 2014). Poly I:C is an agonist of Toll-like receptor (TLR)3, a pattern recognition receptor that allows myeloid cells, including microglia in the brain, to detect local changes in homeostasis by recognizing double-stranded viral RNA (Alexopoulou et al., 2001). Important insights into the pathophysiology of schizophrenia were provided by mIA models, involving neuroinflammation, oxidative stress, neuronal dysfunction, neurotransmitter imbalance, and neurogenesis, among others mechanisms (Lanté et al., 2007; Meyer et al., 2008; Bitanihirwe et al., 2010; Mattei et al., 2014; Manitz et al., 2016).

Microglia, immunes cells that are required for brain development, plasticity, and homeostasis (Tian et al., 2017), were also implicated in schizophrenia, based on evidence from both human and animal studies. In human, exacerbated inflammation and microglial reactivity were reported in patients with schizophrenia, or individuals at ultra-high risk of psychosis (Müller et al., 2015; Najjar and Pearlman, 2015; Bloomfield et al., 2016; Laskaris et al., 2016). In a Poly I:C mouse model, microglia were found to show an increased density as well as reduced process arborization (Juckel et al., 2011), while in a Poly I:C rat model, microglia released increased levels of pro-inflammatory IL-1β and TNFα, and displayed reduced phagocytic activity *ex vivo* (Mattei et al., 2014, 2017). These microglial changes and associated impairments of adult hippocampal neurogenesis and sensorimotor gating in rats were rescued by treatment with the tetracycline derivative minocycline (Mattei et al., 2014, 2017). In a two-hit mouse model combining prenatal Poly I:C with peripubertal stress, microglial alterations, and behavioral abnormalities were similarly normalized by pre-treatment with minocycline (Giovanoli et al., 2016a). Clinical studies have further reported a significant decrease of positive and negative symptoms in schizophrenia patients that received minocycline as an add-on treatment to antipsychotics (Miyaoka et al., 2008, 2012; Levkovitz et al., 2010).

Considering the sex differences in microglial density and morphology described during early postnatal development, as well as in maturation, immune reactivity, and physiological functions across postnatal development, adolescence, and adulthood (Schwarz et al., 2012; Bolton et al., 2014, 2017; Hanamsagar et al., 2017), we hypothesized that microglia could be crucial determinants of sex differences in schizophrenia. In the present study, the effects of mIA on microglia (density, distribution, morphology, ultrastructure), behavior, inflammation, and oxidative stress were compared between male and female offspring at adulthood. Poly I:C was injected into pregnant C57BL/6 mice at embryonic day (E)9.5 (Meyer et al., 2008; Hsiao et al., 2012; Khan et al., 2014; Zhu et al., 2014; Giovanoli et al., 2016b). The prefrontal cortex, hippocampus, and cerebellum, regions where structural and functional alterations were mainly described in schizophrenia patients (Harrison, 2004; Salgado-Pineda et al., 2007; Picard et al., 2008), were selected for analysis.

Our results revealed increased microglial clustering, reduced arborization area, increased cellular stress, and interactions with synapses in hippocampus of male offspring exposed to prenatal Poly I:C. These microglial alterations were accompanied by impaired sensorimotor gating and anxiety-like behavior, alongside inflammation in whole cerebral cortex and hippocampus. Female offspring instead displayed increased microglial contacts with myelinated axons upon prenatal Poly I:C. Sex differences in microglial density and cell body circularity were additionally observed in hippocampus under steady-state and Poly I:C conditions, while both sexes showed increased microglial process area, together with exacerbated stereotypic behavior and impaired sociability.

## Materials and methods

### Animals

All experiments were approved and performed under the guidelines of Université Laval's animal ethics committees and the Canadian Council on Animal Care's. Animals were housed at 22–25°C under a 12-h light–dark cycle with free access to food and water. Experimental animals were generated through mating of C57BL/6 mice from Charles River (St-Constant, QC, Canada). C57BL/6 intruders were also acquired from Charles River (St-Constant, QC, Canada).

### Experimental groups

Viral infection was simulated by injecting Poly I:C potassium salt dissolved in doubled-distilled water (5 mg/kg; Sigma-Aldrich, P9582, St. Louis, MO, USA) intraperitoneally (i.p.) into pregnant mice at E9.5. The pups were weaned at postnatal day (P)21-P22. A vehicle control group was injected with sterile saline. Two to five animals per sex (combination of saline and Poly I:C challenged) were housed together until the onset of experiments at P60. Sixteen litters were used in total for behavioral testing and post-mortem analyses. No significant difference in weight was observed between saline and Poly I:C challenged animals between P60 and P80. The weight ranges of male and female animals for behavioral experiments were 20–26 and 18–23 g in all groups, respectively. Mice with developmental problems (e.g., eye not opened) were excluded from the studies. The numbers of animals used in each experiment are detailed below.

### Behavioral testing

Tests were performed between 9:00 a.m. and 5:30 p.m. under background noise of ~50 db and light intensity of ~50 lux. All behaviors except marble burying, SHIRPA, and prepulse inhibition (PPI) were recorded with the ANY-maze system (version 4.8, Stoelting, Wood Dale, USA). In total, two cohorts of mice were used for two different sets of paradigms. The first cohort sequentially performed marble burying, open field, novel and spatial object recognition, elevated plus maze, and the three-chambered social interaction test from P60 to P80. In total, 16 saline-exposed animals (8 males and 8 females) and 12 Poly I:C-exposed animals (6 males and 6 females) were used in these tests. The second cohort underwent SHIRPA and PPI from P60 to P70. In total, 17 saline-exposed animals (9 males and 8 females) and 14 Poly I:C-exposed animals (7 males and 7 females) were used in SHIRPA and PPI. Detailed testing procedures can be found in the Supporting methods. SHIRPA showed no significant difference in all the parameters measured upon prenatal Poly I:C (Table S1), indicating that the viral mimic does not induce long-term neurological deficits.

### Fluorescent immunohistochemistry and confocal microscopy

Forty-eight hours after the social interaction test, at P80-P90, 16 saline-exposed animals (8 males and 8 females) and 12 Poly I:C-exposed animals (6 males and 6 females) from the first cohort were anesthetized with ketamine (80 mg/kg)/xylazine (10 mg/kg) (i.p.) and transcardially perfused with ice-cold phosphate buffered saline (PBS). Left-brain hemispheres were fixed in 4% paraformaldehyde (PFA; EMS, Hatfield, PA, USA) at 4°C overnight and cut with a cryostat at 30 μm longitudinally. Longitudinal sections containing Bregma 0.36 to 1.00, based on the stereotaxic atlas of Paxinos and Franklin (4th edition), were processed for immunofluorescence staining and confocal imaging. Sections were incubated in 0.1 M citrate buffer at 90°C during 8–10 min for antigen retrieval. After the slides had cooled down, they were washed and blocked in 10% donkey serum (with 0.3% Triton X-100 in PBS) for 1 h at room temperature. All primary and secondary antibodies were diluted in the same blocking buffer. Sections were incubated with IBA1 antibody (1:1000, #019-19741, Wako) at 4°C overnight, rinsed in PBS, and then with an Alexa Fluor 568 secondary antibody (A10040, Thermo-scientific, Waltham, MA, USA) for 2 h at room temperature. Sections were washed in PBS, counter-stained with DAPI (1:20000, Thermo-scientific), and mounted with anti-fading media (H-1000, Vector Laboratories, Burlington, Ontario, Canada) under a glass coverslip.

Using a Quorum WaveFX Spinning disc confocal microscope, microglial imaging was performed in ventromedial prefrontal cortex, hippocampal dentate gyrus (DG), and cerebellum of five to six mice per experimental group. Z-stacks were acquired at 20x magnification with an ORCA-R2 camera (Hamamatsu, 1344 × 1024 pixels) in two areas covering the ventromedial prefrontal cortex, two areas covering the hippocampal DG, and three areas of cerebellum (one image for vermis and two images for cortex). Each stack contained ~30 slices (1 μm each) and focus stacking was performed using Volocity software (Version 5.4, PerkinElmer, Woodbridge, Ontario, Canada).

### Analyses of microglial density, spacing, clustering, and morphology

Quantitative analysis was conducted to assess the density, spacing, clustering, and morphology of microglia in all the images. The analysis was performed blind to the experimental conditions with ImageJ software (National Institutes of Health) as previously described (Tremblay et al., 2012; Milior et al., 2016). To determine cellular density and spacing, the center of each microglial cell body was marked with a dot using the paintbrush tool. The “analyze particles” function was used to automatically record cell numbers and spatial coordinates, in order to determine the nearest neighbor distance for each cell with the “nearest neighbor distance” plugin. Cellular density was determined by dividing the total number of cells by the total surface area of the acquired pictures measured in mm^2^ for each animal. A spacing index was calculated as the square of the average nearest neighbor distance multiplied by microglial density per animal. Clusters comprising two and more microglial cells closer than 12 μm one from another were counted. A morphological index was calculated using the formula: soma area/arborization area. The larger the value, the greater the soma size was in relation to the arbor size. To analyze morphology, a total of 10–15 microglial cells per animal were analyzed. Every IBA1-immunopositive microglia in a particular picture was analyzed before moving on to the next picture as to not introduce selection bias. For each microglia, the soma area was determined by drawing a line around the cell body by using the freehand selection tool. The arborization area was determined with the polygon selection tool to connect the most distal extremities of each process. Soma and arborization areas were calculated in pixels and converted into micrometers. Cell body circularity was determined using the “shape descriptors” measurement tool in Image J and was expressed in arbitrary units. The observer was blinded to the experimental conditions throughout the analysis.

### Molecular analyses using quantitative real-time PCR

The right hemispheres from five animals per experimental group of the first cohort were dissected into cerebral cortex, hippocampus, and cerebellum regions. Tissue was homogenized in QIAzol lysis reagent (#79306, Qiagen, Hilden, Germany) and total RNA was extracted according to the manufacturer's protocol. Subsequently, 1 μg of total RNA was reverse transcribed into cDNA using the iScript cDNA synthesis kit (#170-8891, BioRad, Hercules, CA, USA). Real-time PCR was performed with the SsoAdvanced universal SYBR Green supermix kit (BioRad) in a Lightcycler 480II (Roche, Basel, Switzerland). The sets of primers used are listed in Table S2. Relative expression was calculated with the 2^−ΔΔ*CT*^ method using *Gapdh* for normalization as previously described (Yuan et al., 2006).

### Tissue preparation and immunoperoxidase staining for electron microscopy

For electron microscopy, a separate cohort of three males and three females per experimental group (either exposed to saline or Poly I:C at E9.5) was anesthetized with sodium pentobarbital (80 mg/kg, i.p.) and perfused with 3.5% acrolein and 4% PFA (Bisht et al., 2016a) at P80-P90. Fifty-micrometer thick transverse sections from Bregma 2.12–1.64, based on the stereotaxic atlas of Paxinos and Franklin (4th edition), cut with a vibratome, were processed as previously described (Bisht et al., 2016b). Briefly, transverse sections were washed in PBS, quenched, and processed for IBA1 immunostaining. They were blocked and incubated overnight in primary antibody, incubated with goat anti-rabbit secondary antibody conjugated to biotin (#111-065-003, Jackson ImmunoResearch, West Grove, PA, USA) for 1.5 h, and then with ABC Vectastain (1:100, Vector Laboratories, #PK-6100), followed by diaminobenzidine (0.05%) and hydrogen peroxide (0.015%). The sections were post-fixed in 1% osmium tetroxide, dehydrated in ethanol, and embedded with Durcupan resin between ACLAR films (EMS) at 55°C for 72 h. Areas of interest were cut at 65–80 nm using an ultramicrotome (Leica Ultracut UC7). Ultrathin sections were collected on mesh grids and examined at 80 kV with a FEI Tecnai Spirit G2 transmission electron microscope.

### Analyses of microglial ultrastructural features

Ultrastructural observations were conducted at the tissue–resin border, where the penetration of antibodies and staining intensity is maximal (Tremblay et al., 2010b). Profiles of neurons, synaptic elements, microglia, astrocytes, oligodendrocytes, and myelinated axons were identified according to established criteria (Peters et al., 1991). Microglia showing well-characterized signs of oxidative stress, including condensed, electron-dense cytoplasm and nucleoplasm, cytoplasmic shrinkage, dilated Golgi apparatus and endoplasmic reticulum, as well as mitochondrial alteration, and very weak immunostaining for IBA1 were referred to as “dark” microglia (Bisht et al., 2016b). To measure the density of dark microglia, the square-mesh grids were sequentially imaged at the lowest magnification (440x) under our microscope to systematically determine the total number of grid squares enclosing neuropil tissue from the DG polymorphic layer. Its total surface area was calculated at high precision by multiplying the number of grid squares containing that layer by the area of a single grid square, as previously explained in details (Bisht et al., 2016b). A schematic representation of all grid squares included in the analysis was drawn for each animal. The grid squares were afterward rigorously screened for the presence of dark microglia as previously described (Bisht et al., 2016b). Dark microglia's density was expressed as numbers per mm^2^ of tissue surface. The analysis was performed blind to the experimental conditions. For quantitative analysis of IBA1-stained processes from “typical” microglia, ~75 profiles per animal were randomly captured at 6,800x using an ORCA-HR digital camera (Hamamatsu; 10 MP). The area, perimeter, and shape descriptors “circularity” and “solidity” were used to assess changes in morphology with ImageJ. Direct contacts with synaptic clefts and myelinated axons were counted for each microglial process profile. Vacuoles associated with autophagy or phagocytosis, and endosomes containing cellular materials in the process of being digested (termed “cellular” inclusions) were also counted on a microglial process profile basis (Tremblay et al., 2010a). The analysis was performed blind to the experimental conditions.

### Statistics

Data were analyzed using Prism (GraphPad, Version 5). Two-way ANOVAs with Bonferroni *post-hoc* tests were used to determine interactions between Poly I:C exposure and sex effects in all groups unless otherwise specified. An online Grubbs' test calculator (GraphPad Software, https://www.graphpad.com/quickcalcs/Grubbs1.cfm) was used to determine significant outliers in all experiments and the outliers were removed from the datasets. Sample size (*n*) refers to animals in all experiments, except for the ultrastructural analyses of IBA1-immunopositive microglial processes where it refers to individual profiles as previously published by our group (Milior et al., 2016). *p* < 0.05 was considered statistically significant. All reported values are mean ± standard error of the mean (S.E.M.).

## Results

### Poly I:C alters microglial distribution and morphology, especially in male hippocampus

The combined findings from previous studies demonstrate various changes in microglial density, morphology, phagocytic activity, gene and protein expression within several brain regions of Poly I:C-challenged rodent models (Ribeiro et al., 2013; Mattei et al., 2014, 2017; Van den Eynde et al., 2014; Zhu et al., 2014; Eßlinger et al., 2016; Manitz et al., 2016) and schizophrenia patients (Müller et al., 2015; Najjar and Pearlman, 2015; Bloomfield et al., 2016; Laskaris et al., 2016). Also, prenatal Poly I:C treatment at E9 has been shown to suppress the effects of environmental enrichment on the increase of microglial density in mouse cerebral cortex and hippocampus during adulthood (Buschert et al., 2016). However, unaltered microglial density and morphology were also reported among the DG and hippocampus CA1 of adult mice exposed to a prenatal Poly I:C challenge (Giovanoli et al., 2015, 2016b). To study further microglial alterations and their possible sex-differences induced by prenatal Poly I:C, extensive morphological analyses were performed in male vs. female adult (P80-P90) offspring exposed to Poly I:C at E9.5. The prefrontal cortex, DG, and cerebellum, associated with structural and functional alterations in schizophrenia patients, were examined.

**Figure 1:**
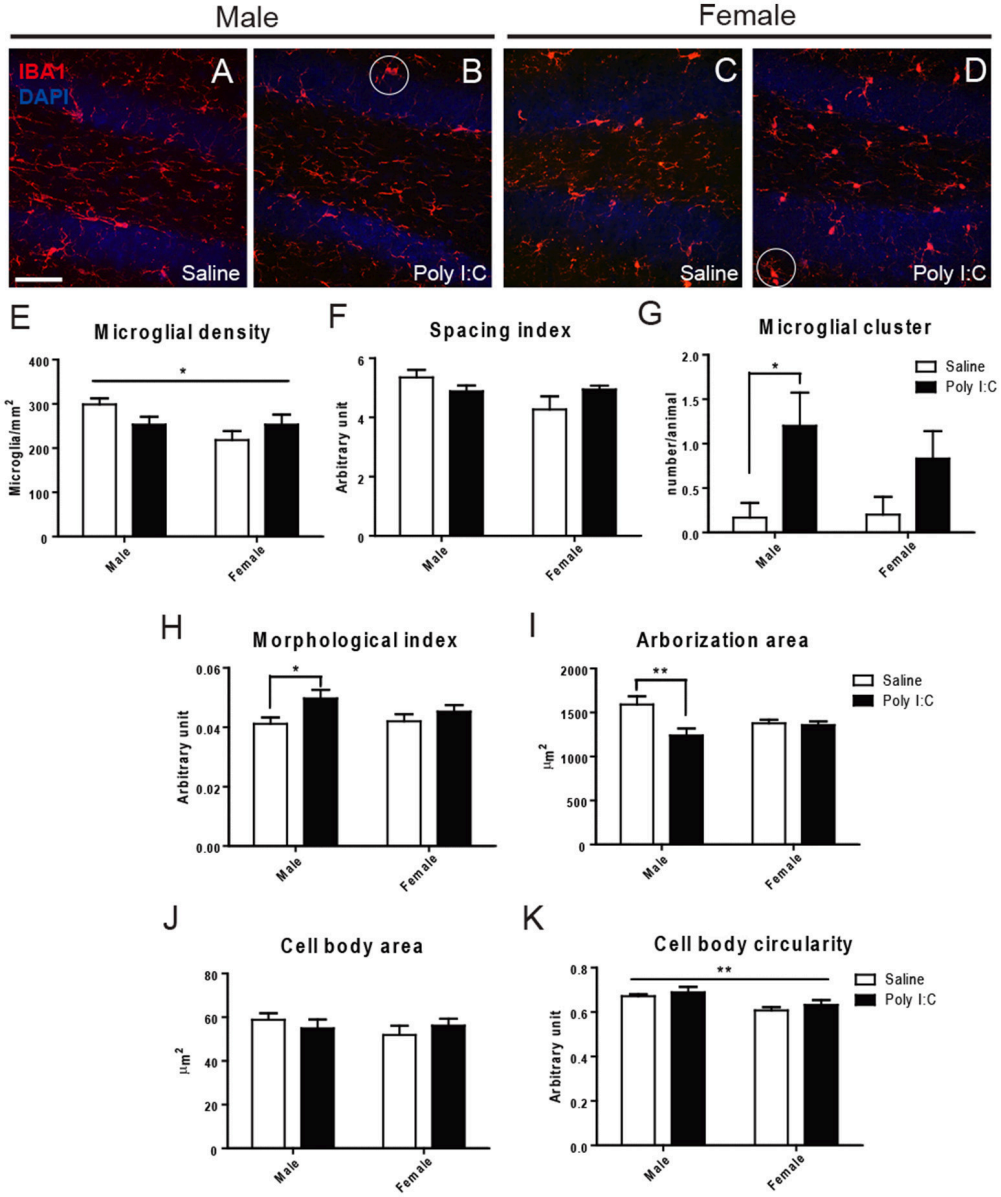
Effects of Poly I:C on microglial distribution and morphology in hippocampal DG. **(A–D)** Low magnification (20x) pictures showing the density of IBA1-stained microglial cells. Examples of clustering are encircled in **(B,D)**. **(E–K)** Microglial density, spacing index, clustering, morphological index, arborization area, cell body area, and cell body circularity are shown. In **(E,K)**, the asterisk refers to comparison between sexes. *n* = 5–6 mice per sex and group. Scale bar: 50 μm. ^*^p < 0.05, ^**^p < 0.01.

Microglial density, spacing index, cell body area, and cell body circularity were relatively unchanged across experimental groups, in the three examined regions (Figure 1 and Figures S1, S2). However, ANOVA showed a significant Poly I:C x sex interaction for spacing index in the DG [*F*_(1, 18)_ = 4.45, *p* = 0.0491] (Figure 1F). ANOVA also showed a main effect of Poly I:C on microglial arborization area [*F*_(1, 18)_ = 7.10, *p* = 0.0158], with significant Poly I:C x sex interaction [*F*_(1, 18)_ = 5.73, *p* = 0.0278] in DG, while *post-hoc* analysis revealed a reduced arborization area in Poly I:C-exposed males only (*p* < 0.01, Figure 1I). When a morphological index was computed by dividing soma area by arborization area (Tremblay et al., 2012), a ratio which is susceptible to increase in reactive microglia (Streit et al., 1999), a main effect of Poly I:C was identified in the DG [*F*_(1, 18)_ = 5.94, *p* = 0.0254], without Poly I:C x sex interaction. In addition, *post-hoc* analysis revealed that the increase was significant in Poly I:C challenged males only (*p* < 0.05, Figure 1H). Sex differences in microglial distribution and morphology were further identified, with a significant effect of sex on microglial density [*F*_(1, 18)_ = 4.54, *p* = 0.0472] (Figure 1E) and cell body circularity [*F*_(1, 18)_ = 10.71, *p* = 0.0042] in the DG (Figure 1K). An increased clustering of microglia, in which neighbor cells lose their territorial organization and become close to one another, was also identified in animals exposed to Poly I:C (Figures 1B,D). ANOVA showed a main effect of Poly I:C on the number of clusters in the DG [*F*_(1, 18)_ = 4.54, *p* = 0.0472], without Poly I:C x sex interaction, and *post-hoc* analysis indicated that the change occurred in males selectively (*p* < 0.05, Figure 1G).

### Poly I:C increases dark microglia's density in males, while inducing sex-differences in typical microglia

**Figure 2:**
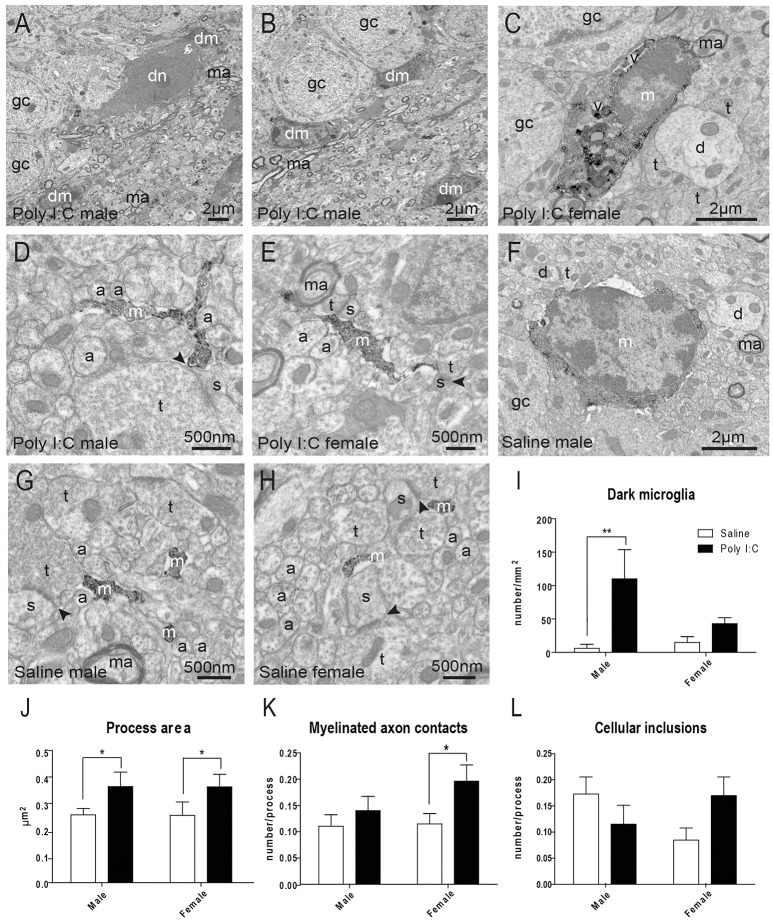
Effects of Poly I:C on microglial ultrastructure in hippocampal DG. **(A–E)** Examples of cell bodies and processes from dark microglia (dm) vs. typical microglia (m) that show strong immunoreactivity for IBA1 and directly contact dark neurons (dn) with features of cellular stress. **(F–H)** Examples of IBA1-stained microglial cell body and processes in saline-exposed offspring of both sexes. **(I)** Quantification of dark microglia's density in the polymorphic layer. **(J–L)** Microglial process area, number of contacts with myelinated axons (ma), and number of cellular inclusions in the polymorphic layer. *n* = 3 animals (dark microglia) or 224–296 profiles (IBA1-stained processes) in three mice per sex and group. Arrowhead, synaptic cleft; a, unmyelinated axon; d, dendritic spine; gc, granule cell; t, axon terminal; v, vacuole. ^*^p < 0.05, ^**^p < 0.01.

In the adult offspring (P80-P90) exposed to prenatal Poly I:C, we subsequently conducted EM analyses to determine the density of “dark” microglia, a newly-defined phenotype that displays several ultrastructural features of cellular stress, weak immunoreactivity for IBA1, extensive contacts with pre- and post-synaptic elements, as well as strong immunoreactivity for CD11b in processes encircling synaptic elements (Bisht et al., 2016b) (Figures 2A,B). CD11b is an essential component of complement receptor 3 mediating microglial pruning of axon terminals (Schafer et al., 2012). ANOVA first revealed a main effect of Poly I:C on the density of dark microglia [*F*_(1, 8)_ = 8.37, *p* = 0.0201], without significant Poly I:C x sex interaction, and *post-hoc* analysis identified a selective increase in males (*p* < 0.01, Figure 2F).

Typical microglia, which are strongly immunoreactive for IBA1 and focally contact (rather than encircle) synaptic elements, were also analyzed (Tremblay et al., 2010a; Bisht et al., 2016b) (Figures 2C–H). ANOVA identified a main effect of Poly I:C on microglial process area [*F*_(1, 977)_ = 5.14, *p* = 0.0236], perimeter [*F*_(1, 977)_ = 6.63, *p* = 0.0102], and contacts with myelinated axons [*F*_(1, 977)_ = 5.02, *p* = 0.0253]. Poly I:C x sex interaction was not significant for microglial process area, perimeter, and contacts with myelinated axons. However, the increase of perimeter and contacts with myelinated axons were found to be significant in Poly I:C-treated female offspring after *post-hoc* analysis (both *p* < 0.05, Figure 2K and Figure S3A), without any sex difference for the increase of process area (Figure 2J). Interestingly, ANOVA also revealed a significant sex x Poly I:C interaction for the number of cellular inclusions within microglial processes [*F*_(1, 977)_ = 5.10, *p* = 0.0242], with Poly I:C resulting in opposite trends between the sexes (Figure 2L). Microglial process circularity, contacts with synaptic clefts, and number of vacuoles were unchanged (Figures S3B–D).

### Poly I:C-induced microglial alterations are accompanied by behavioral deficits in males

To characterize sex differences in behavioral outcome associated with microglial alterations, the offspring having received Poly I:C challenge at E9.5 was subjected during adulthood (P60-P90) to extensive testing of motor control, anxiety, sociability, memory, and sensorimotor gating.

**Figure 3:**
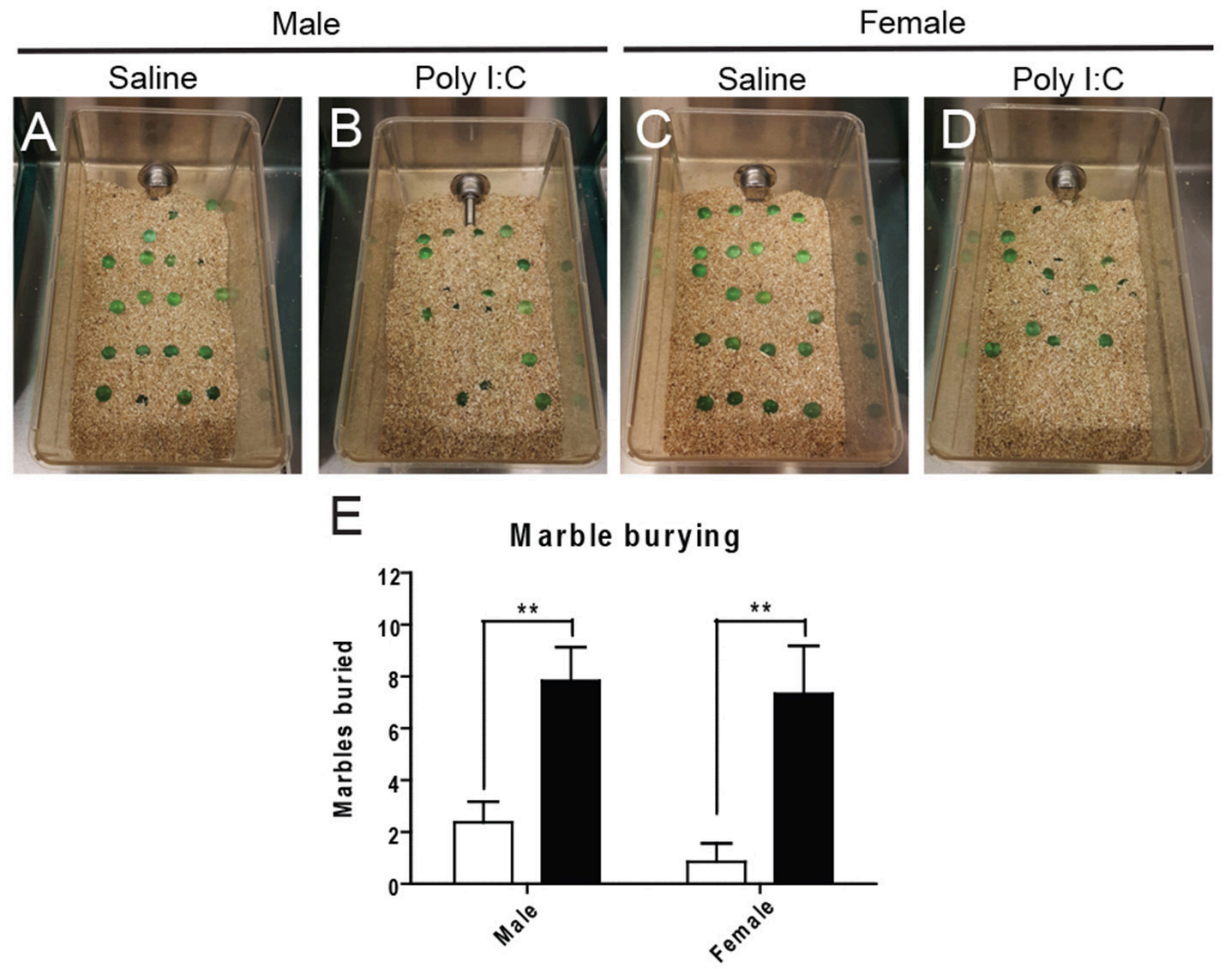
Effects of Poly I:C on repetitive behaviors. Representative pictures of the experimental cages after marble burying are shown in **(A–D)**. Numbers of marbles buried as quantified in **(E)**. *n* = 6–8 mice per sex and group. ^**^p < 0.01.

Marble burying was first performed to distinguish abnormal repetitive behavior (see Figures 3A–D for representative images). ANOVA revealed a significant effect of Poly I:C [*F*_(1, 23)_ = 25.84, *p* < 0.0001] and *post-hoc* analysis showed that adult offspring from both sexes display increased repetitive behavior after prenatal Poly I:C exposure (*p* < 0.01, Figure 3E).

**Figure 4:**
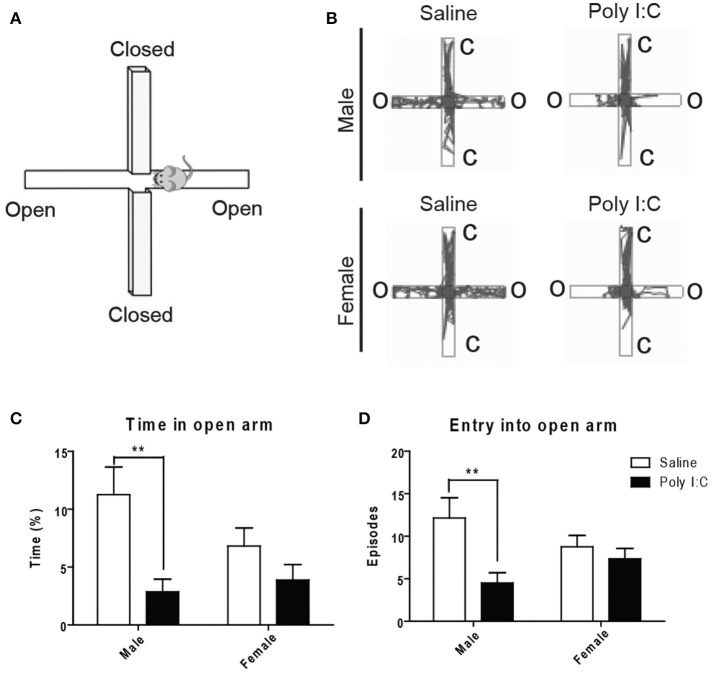
Effects of Poly I:C on anxiety-like behavior measured in the elevated plus maze. **(A)** Orientation of the elevated plus maze platform. **(B)** Representative motion plots in males and females from saline and Poly I:C injection groups. **(C,D)** Anxiety level was determined by measuring the time spent and number of entries in the open arm during testing. *n* = 6–8 mice per sex and group. C, closed arm; O, open arm. ^**^p < 0.01.

With the open field (see Figures S4A,B for representative images), no changes in locomotion or exploratory activity were measured in the Poly I:C-exposed offspring at adulthood (Figures S4C–F). However, ANOVA showed main effects of Poly I:C [*F*_(1, 23)_ = 12.02, *p* = 0.0027] and sex [*F*_(1, 23)_ = 7.39, *p* = 0.0141] on anxiety-like behavior, in males only, based on their dropping of significantly more fecal boli (*post-hoc* analysis; *p* < 0.05, Figures S4G–I). To measure anxiety more selectively, the animals were afterwards tested with the elevated plus maze (Figure 4A). ANOVA showed a main effect of Poly I:C on the time spent [*F*_(1, 23)_ = 10.52, *p* = 0.0036] and number of entries in the open arms [*F*_(1, 23)_ = 7.32, *p* = 0.0126]. Although Poly I:C x sex interactions were not significant, *post-hoc* analysis revealed that adult male offspring exposed to prenatal Poly I:C spent significantly less time (*p* < 0.01, Figure 4C) and entered the open arms less frequently (*p* < 0.01, Figure 4D) than male offspring receiving saline, as shown in the representative plots (Figure 4B), thus indicating enhanced anxiety-like behavior. No changes in time spent in closed arms, entry into closed arms, total distance traveled, and total immobile time were identified between groups (Figures S5A–D).

**Figure 5:**
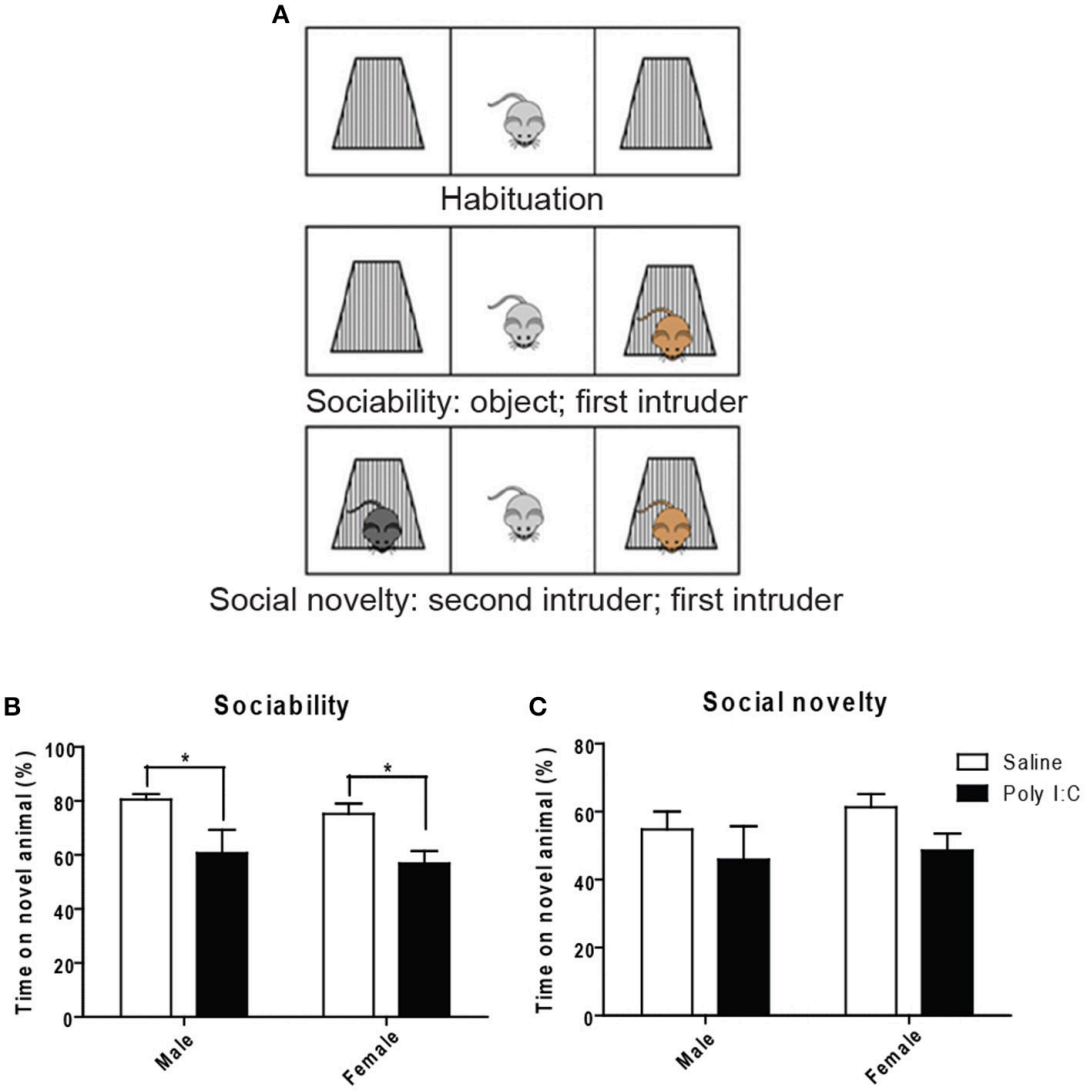
Effects of Poly I:C on sociability and social novelty assessed with the three-chambered social interaction test. **(A)** Schematic representation of the procedure for testing (modified from http://med.stanford.edu). Quantification of sociability is shown in **(B)** and social novelty is shown in **(C)**. *n* = 6–8 mice per sex and group. ^*^p < 0.05.

Using the three-chamber social interaction test (Figure 5A), ANOVA showed a main effect of Poly I:C on sociability [*F*_(1, 23)_ = 14.35, *p* = 0.0010], measured as the propensity to interact with a novel intruder, without Poly I:C x sex interaction. Similar to our findings with marble burying, *post-hoc* analysis revealed that Poly I:C reduces sociability in offspring from both sexes (*p* < 0.05, Figure 5B). Also, social novelty (or social memory), defined as the ability to recognize a second intruder, was not affected by prenatal Poly I:C (Figure 5C).

To further characterize our mIA model, novel and spatial object recognition memory was next assessed (Figure S6A). However, no deficit in novelty or spatial memory was observed in the adult offspring exposed to prenatal Poly I:C (Figures S6B,C).

**Figure 6:**
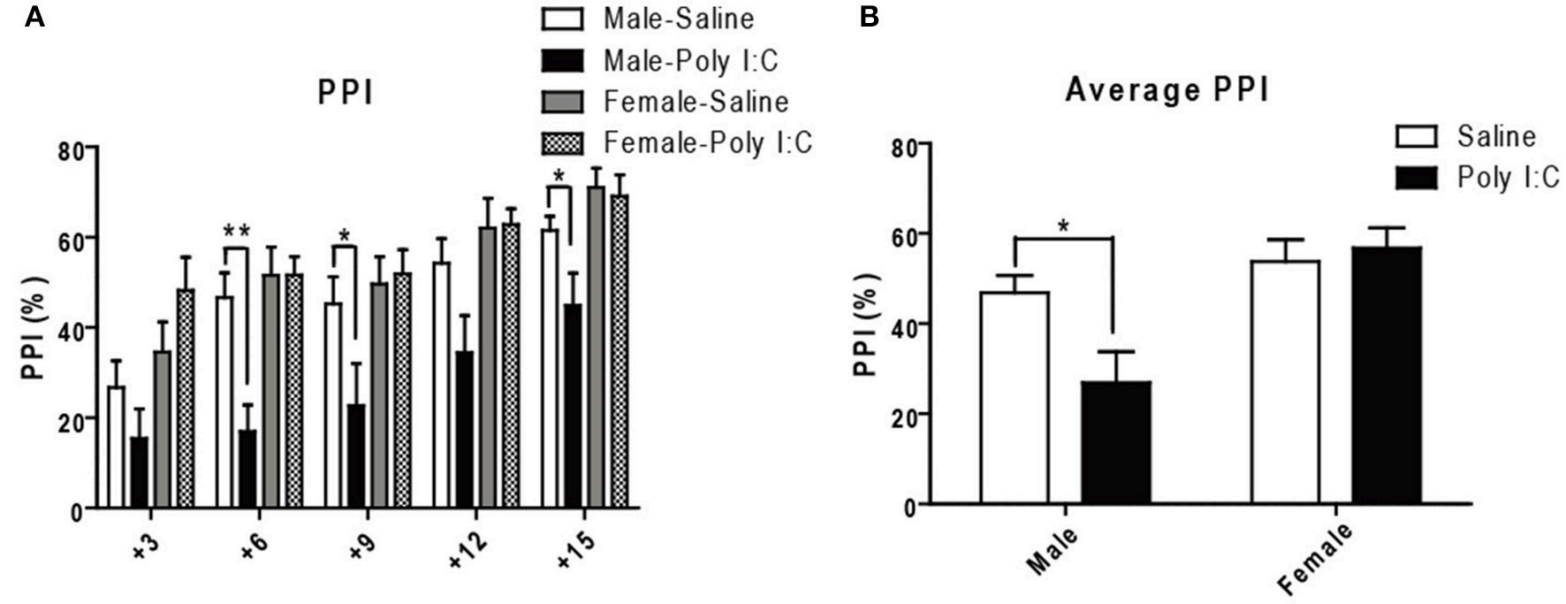
Effects of Poly I:C on sensorimotor gating activity under prepulse inhibition paradigm. **(A)** Sensorimotor gating was determined by calculating percentage reduction of startle amplitude using five prepulses (3–15 dB higher than background noise) administered before the 120 dB startle tone. **(B)** The average reduction of startle amplitude is shown. *n* = 7–9 mice per sex and group. ^*^p < 0.05, ^**^p < 0.01.

Since PPI is considered a translatable model of sensorimotor deficits consistently measured in schizophrenia patients (Swerdlow et al., 2016), we lastly performed acoustic PPI in adult offspring having received Poly I:C according to the protocol described in the Supporting method. ANOVA showed main effects of Poly I:C on PPI at 76 dB [*F*_(1, 27)_ = 7.06, *p* = 0.0131], and of sex at all the prepulses tested 73 dB:[*F*_(1, 27)_ = 9.38, *p* = 0.0049], 76 dB:[*F*_(1, 27)_ = 12.54, *p* = 0.0015], 79 dB: [*F*_(1, 27)_ = 6.17, *p* = 0.0195], 82 dB: [*F*_(1, 27)_ = 8.59, *p* = 0.0068], 85 dB: [*F*_(1, 27)_ = 8.59, *p* = 0.0068], with Poly I:C x sex interaction showing significance only at 76 dB [*F*_(1, 27)_ = 7.07, *p* = 0.0130]. *Post-hoc* analysis additionally revealed that Poly I:C induces deficits at 76 dB (*p* < 0.01), 79 dB (*p* < 0.05), and 85 dB (*p* < 0.05) only in male offspring (Figure 6A). After averaging PPI values from all the prepulse-pulse trials (Figure 6B), ANOVA further showed main effects of sex [*F*_(1, 27)_ = 13.50, *p* = 0.0010] with significant Poly I:C x sex interaction [*F*_(1, 27)_ = 5.26, *p* = 0.0299]. *Post-hoc* analysis lastly revealed a significant reduction of PPI in male offspring after Poly I:C exposure (*P* < 0.05, Figure 6B), indicating that Poly I:C may result in this schizophrenia-like behavior specifically in males.

### Poly I:C also exacerbates inflammation in male cerebral cortex and hippocampus

To provide molecular insights into the sex differences in microglia and behavior, qRT-PCR was conducted in cerebral cortex, hippocampus and cerebellum of male and female adult offspring (P80-P90) quantifying expression levels of genes related to inflammation, oxidative stress, and microglial homeostatic phenotype that is altered in the human disease (Chen et al., 2012; Ferretti et al., 2014; Colonna and Wang, 2016; López González et al., 2016).

**Figure 7:**
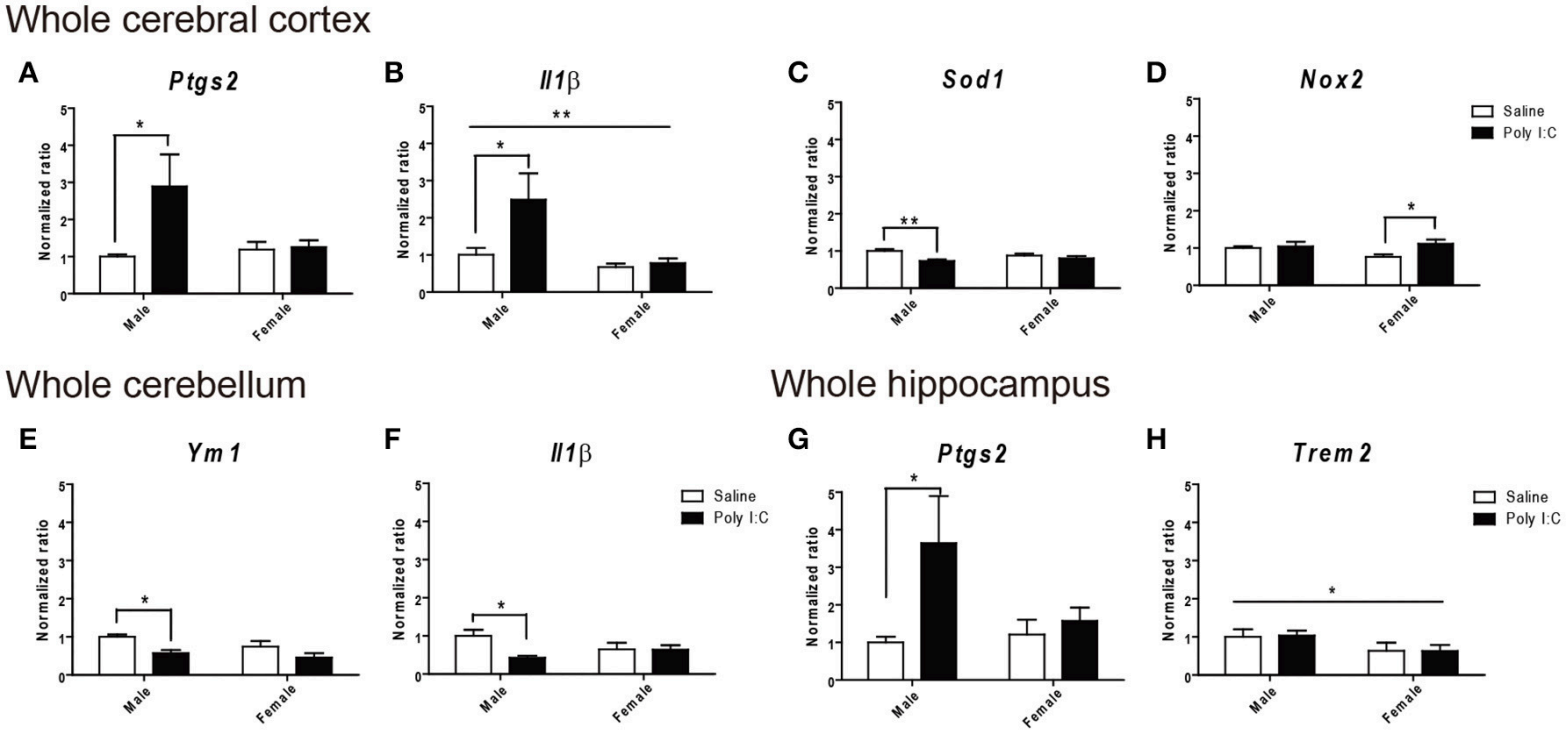
Effects of Poly I:C on gene expression in whole cerebral cortex, cerebellum, and hippocampus. Significant changes in gene expression levels of **(A–D)** *Ptgs2, Il1*β, *Sod2*, and *Nox2* were measured in the cerebral cortex, **(E,F)** *Ym1* and *Il1*β in the cerebellum, and **(G–H)** *Ptgs2* and *Trem2* in the hippocampus. *n* = 4–5 mice per sex and group. ^*^p < 0.05, ^**^p < 0.01.

Gene expression ratios are provided in Figure 7 and Tables S3–S5. In the whole cerebral cortex, ANOVA identified main effects of Poly I:C on *Sod1* [*F*_(1, 14)_ = 10.28, *p* = 0.0063], *Ptgs2* coding for COX-2 protein [*F*_(1, 16)_ = 4.66, *p* = 0.0465], and *Il1*β [*F*_(1, 14)_ = 5.71, *p* = 0.0316], and of sex on *Il1*β [*F*_(1, 8)_ = 6.69, *p* = 0.0323], and *Nox2* [*F*_(1, 16)_ = 4.56, *p* = 0.0485], without significant Poly I:C x sex interaction identified. In particular, *post-hoc* analysis revealed that Poly I:C significantly increases inflammatory *Ptgs2* (*p* < 0.05) (Figure 7A) and *Il1*β (*p* < 0.05) (Figure 7B) and reduces anti-oxidant *Sod1* (*p* < 0.01) (Figure 7C) in male offspring, while increasing oxidant *Nox2* (*p* < 0.05) (Figure 7D) in female offspring. Consistent with these results in cortex, ANOVA identified a main effect of Poly I:C [*F*_(1, 16)_ = 4.82, *p* = 0.0433] on *Ptgs2* in hippocampus, without Poly I:C x sex interaction, and *post-hoc* analysis indicated that males exposed to Poly I:C are particularly vulnerable, showing increased inflammatory *Ptgs2* in hippocampus (*p* < 0.05) (Figure 7E). A main effect of sex was also identified for the phagocytosis-related gene *Trem2* [*F*_(1, 16)_ = 4.64, *p* = 0.0468] in hippocampus (Figure 7F), which might suggest its contribution to the opposite changes in phagocytosis between sexes (Figure 2I). A main effect of Poly I:C was shown for the anti-inflammatory *Ym1* [*F*_(1, 16)_ = 11.40, *p* = 0.0038] and inflammatory *Il1*β [*F*_(1, 15)_ = 4.98, *p* = 0.0414] in cerebellum, without Poly I:C x sex interaction, and *post-hoc* test showed reduced expression of both genes in males only (*p* < 0.05) (Figures 7G,H). No changes in the homeostatic genes *Cx3cr1* and *Cx3cl1* were identified in all brain regions investigated. These data suggest that prenatal exposure to Poly I:C exacerbates inflammation and induces oxidative imbalance, mainly to the cerebral cortex and hippocampus of males.

## Discussion

The present findings show that Poly I:C-induced prenatal immune challenge at E9.5 triggers behavioral impairments accompanied by exacerbated inflammation, oxidative stress, and microglial alterations, particularly in males. Sex differences in Poly I:C models have remained largely undetermined due to the use of mixed sexes and different timing of exposure or species across studies (Meyer et al., 2008; Mattei et al., 2014; Zhu et al., 2014; Aavani et al., 2015). Our behavioral characterization in the two sexes revealed that male offspring exposed to Poly I:C at E9.5 have additional deficits, compared with females, affecting anxiety and sensorimotor gating, in agreement with the previous human (Aleman et al., 2003; McGrath et al., 2004) and animal studies measuring sensorimotor gating and sociability (Bitanihirwe et al., 2010; Xuan and Hampson, 2014).

In our analyses, pro-inflammatory gene levels were found to increase in the cerebral cortex and hippocampus of male offspring exposed to Poly I:C at E9.5, indicating long-lasting effects of prenatal infection into adulthood. Chronic inflammation has been reported in schizophrenia patients and mIA models (Garay et al., 2013; Khandaker et al., 2013; Mattei et al., 2014, 2017) and was associated with pronounced behavioral impairment (Cunningham et al., 2009; Mattei et al., 2014, 2017; Harrison et al., 2015; Müller et al., 2015). The sex-dependent increase of inflammation that we measured in the hippocampus of Poly I:C-challenged male offspring is consistent with previous rodent studies reporting sex differences in microglial reactivity upon immune challenge (Ho et al., 2015; Hanamsagar et al., 2017) and during aging (Mangold et al., 2017). While oxidative stress was proposed to underlie the social memory and neurocognitive deficits of schizophrenia patients and mIA models (Bošković et al., 2011; Flatow et al., 2013; Emiliani et al., 2014; Gonzalez-Liencres et al., 2014; Hardingham and Do, 2016), our gene expression analysis suggested an increase in the cerebral cortex of males receiving Poly I:C challenge.

In line with the recent findings of sex differences in microglial density, especially of amoeboid cells, reported in DG during early postnatal development (Schwarz et al., 2012; Bolton et al., 2014, 2017; Laskaris et al., 2016; Hanamsagar et al., 2017), in our analyses, reduced arborization area was measured in DG of adult male offspring after E9.5 Poly I:C challenge. Reduced number of microglial processes was previously reported at adulthood in DG of another schizophrenia model, the male Gunn rat (Liaury et al., 2012), suggesting altered surveillance of the brain parenchyma. A recent study by Bolton and colleagues revealed that microglia in DG display enlarged cell bodies with thinner and longer processes in P30 male mice that were prenatally-challenged by air pollution, suggesting that microglia in this hippocampal region are primed after the prenatal immune challenge (Bolton et al., 2017). Consistent with this finding, Bolton and colleagues had previously shown that high fat diet consumption during adulthood increases gene expression levels of the microglia/macrophage markers *Cd11b, Cx3cr1*, and *Tlr4* in hippocampus of male offspring prenatally-challenged by air pollution (Bolton et al., 2014). In our study, cell body area remained unchanged across experimental groups, while cell body circularity was decreased in females under both Poly I:C and steady-state conditions. We also found microglial clusters that were more prevalent in the DG of males exposed to Poly I:C at E9.5. Microglial clustering without concomitant increase in density—a feature which however decreased in females under both Poly I:C and steady-state conditions—indicates the occurrence of neuropil areas unsampled by microglia, which could result in impaired response to damage. Similarly, microglial density was previously shown to remain unaltered upon E9 Poly I:C challenge, when measured at adulthood, in the DG of male CD1 mice (Buschert et al., 2016). These microglial clusters also raise the intriguing possibility of self-renewing microglia locally proliferating after Poly I:C stimulation (Bruttger et al., 2015).

Ultrastructural analyses further revealed a significant increase in the density of dark microglia extensively interacting with synapses in DG of males receiving Poly I:C stimulus. These cells dowregulating IBA1 were recently found to be associated with pathological states and to display well-characterized signs of cellular stress (Bisht et al., 2016b). This finding suggests that dark microglia could remodel neuronal circuits in a dysfunctional manner in schizophrenia, a hypothesis that warrants further investigation. Processes from typical microglia strongly positive for IBA1 also showed an increased area, in both sexes, supporting the process thickening that was previously reported in Gunn rat (Liaury et al., 2012). In females, unexpectedly, not only were microglial process area and perimeter increased, but also their number of contacts with myelinated axons, which may indicate their involvement with the removal of injured axons (Lafrenaye, 2016). Microglial phagocytic activity was additionally assessed by counting the number of cellular inclusions per microglial processes. In our model, Poly I:C challenged males showed a trend toward reduced phagocytic activity (less cellular inclusions), which is consistent with previous studies (Mattei et al., 2014, 2017). Females displayed an opposite trend (more cellular inclusions) after Poly I:C challenge, supporting the differential microglial responses to prenatal infection between sexes. Whether all of these microglial ultrastructural changes are beneficial or detrimental, and underlying the behavioral alterations, requires further investigation.

These changes were observed in the DG, an hippocampal region that is involved in learning and contributes to spatial and episodic memory by separating activity patterns in the entorhinal cortex, and encoding a large number of inputs (Leutgeb et al., 2007; Bakker et al., 2008). It is also one of the main regions where neurogenesis persists in the adult brain (Sierra et al., 2014). Computational models and human studies identified a pattern separation deficiency in schizophrenia patients suggesting that DG dysfunction (Das et al., 2014; Faghihi and Moustafa, 2015; Martinelli and Shergill, 2015) might underlie their impairment of declarative memory (Das et al., 2014). The proposed mechanisms include a high enrichment of *Tmem108*, a schizophrenia-susceptibility gene, in DG granule cells which results in impaired glutamatergic transmission in mice (Jiao et al., 2017), the reduction of glutamate signaling measured specifically in DG of schizophrenia patients (Stan et al., 2015), and alteration of adult hippocampal neurogenesis that was reported both in animal models and patients (Reif et al., 2006; Walton et al., 2012). These findings suggest that the DG is a region primarily affected in schizophrenia progression and we hypothesize that microglial alterations in the DG may represent one of the factors leading to its dysfunction in schizophrenia patients. Further insights into the pathophysiological mechanisms will be provided by investigating how compromised neuron-microglia interactions in DG result in sex-dependent behavioral deficits.

Overall, our results indicate that schizophrenia's higher incidence in males might be associated, among other mechanisms, with an increased microglial reactivity to prenatal immune challenges. Additional studies are warranted to elucidate, in both sexes, microglial implication with the exacerbated inflammation and oxidative stress, the pathological remodeling of neuronal circuits, and behavioral impairments in schizophrenia and other neuropsychiatric disorders associated with prenatal immune challenges.

## Author contributions

CWH and M-ÈT: designed the study; CWH, AS-P, HEH, YR, and M-ÈT: conducted experiments and analyzed data; SSH, GNL, and LS: provided expertise and resources; CWH, GNL, LS, and M-ÈT wrote the manuscript.

### Conflict of interest statement

The authors declare that the research was conducted in the absence of any commercial or financial relationships that could be construed as a potential conflict of interest.
