# Supplementary material for: Prenatal Immune Challenge in Mice Leads to Partly Sex-Dependent Behavioral, Microglial, and Molecular Abnormalities Associated with Schizophrenia
Source: Front Mol Neurosci. 2018 Feb 8;11:13. doi: 10.3389/fnmol.2018.00013 (PMC5809492; doi:10.3389/fnmol.2018.00013)
Supplement: Supplementary file 1 [file DataSheet1.pdf]

Supporting information

Supporting methods

Marble burying

The mice were handled gently two to three days prior to testing to reduce psychological stress. Marble burying, a test used to determine repetitive behavior in rodents (Thomas et al., 2009), was performed in a clean standard cage (12cm x 25cm). Fresh unscented bedding material made of sawdust was added to a depth of 4.5 to 5cm. Twenty marbles (1.5cm in diameter) of green color (to increase discrimination from the bedding) were placed in the cages using a 4 x 5 distribution. Each mouse was introduced in a cage, allowed screening for 30 min, and then returned to its home cage. Marbles were counted as buried when the bedding covered 75% of their surface. An observer blinded to the experimental conditions counted the number of buried marbles manually.

Open field

The open field test was used to determine locomotor activity, exploration habits, emotional stress, and anxiety. Assessment took place within a square arena (50 x 50cm, made of white cardboard) in which the center zone (25 x 25cm) was delineated by the ANY-maze system (Stoelting Co.). Each mouse was individually placed in the center of the square arena and allowed free movement for 10 min while being tracked by the ANY-maze system. The total distance travelled, number of line crossing, time spent in central and peripheral areas, and other behaviors (immobility, freezing, body rotation, and jumping) were automatically recorded. At the end of testing, for each animal, the number of feces was also counted to assess emotional stress. The open field platform was cleaned with 70% ethanol between animals.

Novel and spatial object recognition

The recognition paradigm, which tests novelty and spatial memories (Antunes and Biala, 2012;Goh and Manahan-Vaughan, 2013), was performed with the open field platform. Each mouse was individually placed in the center of the square arena for 10 min of habituation. After 24 hours, the mice were adapted to two identical silver cubes (5 x 5 x 5cm). After 1 hour, they were tested for either novel object or spatial object recognition for 10 min. For novel object recognition, one of the cubes was replaced by a golden truncated pyramid (5 x 5 x 4.5cm). For spatial object recognition, one of the cubes was moved to another location. The tests were

performed on two separate days. All objects used were made from steel without odor and with protection against corrosion. The time for approaching each object was recorded by the ANY-maze system and the time of interaction with novel objects calculated. The open field platform was cleaned with 70% ethanol between animals.

Elevated plus maze

Elevated plus maze was performed to measure anxiety in a more sensitive manner. The apparatus for elevated plus maze consisted of a cross chamber (made of laminated cardboard), with two open arms and two closed arms surrounded by walls. Each arm measured 5cm x 30cm. The whole apparatus was elevated to 30cm. Each mouse was placed in the center of the chamber, with its head facing the upper closed arm, and was allowed to freely explore the arms for 10 min. For each tested animal, the number of arm entries, distance travelled, and time spent in the open and closed arms were recorded by the ANY-maze system. The elevated plus maze was cleaned with 70% ethanol between animals.

Three-chambered social interaction

Sociability and social novelty were measured with the three-chambered social interaction method of Crawley (Kaidanovich-Beilin et al., 2011). The apparatus consisted of a rectangular box in which each chamber measured 20cm x 40cm. Walls were made of transparent Plexiglas. Small open doors between the three chambers allowed for free circulation of the animals across chambers. Two identical, vertically placed, and cup-like containers, large enough to hold a single mouse, were used. The containers, one in each side chamber, served to enclose the intruder (unfamiliar mouse of the same strain, sex and age as the tested animal). During habituation, the animals were placed in the middle chamber and allowed to explore the area for 10 min. After cleaning with 70% ethanol, an intruder was placed in one of the containers to test for sociability. The tested animal was allowed to search and interact with the intruder for 10 min. After cleaning the chamber with soap water, a second intruder was placed in the other container to assess social novelty and the tested animal was allowed to interact with both intruders for another 10 min. The interaction time with each intruder and total distance traveled of the tested animal were measured by the ANY-maze system.

RIKEN modified SHIRPA behavioral assessment

Modified SHIRPA (version 4) involved 68 tests used to evaluate morphology, behavior, sensory response, and athletic ability. The detailed version of the SHIRPA protocol and scoring methods are provided on the webpage of RIKEN BioResource Center: http://ja.brc.riken.jp/lab/jmc/shirpa/. Briefly, mice were first evaluated under a transparent viewing jar made of Plexiglas during 5 min for body position, movement type, body position, respiration rate, body tremor, and the presence of fecal boli or urine. The mice were placed in the open field arena (55cm x 33cm) to test for transfer arousal, locomotor activity, piloerection, gait, auditory startle to a 90 db tone provided by a metal clicker, pelvic elevation, tail position, and touch escape. The mice were then lifted by the tail for 30 s and assessed for the position at which struggling movements occurred, as well as trunk curl and limb grasping. After landing on the horizontal grid, the mice were evaluated for grasping, grip strength when dragged by the tail, body tone after finger compression on each side, pinna and corneal reflexes, as well as toe-pinch withdrawal of a hindlimb upon squeezing with a hand-held forceps. After restraint in a supine position, body and tail lengths, eye conditions, skin color, heart rate, hindlimb tone, abdominal tone, lacrimation, salivation, and provoked biting in response to a plastic probe gently put in their mouth were observed. The forepaws of mice were then placed on a horizontal bar and their ability to hang suspended (wire maneuver) was assessed. The drop righting reflex was evaluated according to landing position, followed by contact righting when placed inside of a small plastic tube. In the final phase, the mice were placed on a horizontal grid rotated towards a vertical position and assessed for negative geotaxis. Neurological signs, such as abnormal reflexes, notably limb-clasping or a bat-like posture, and seizure activity, were recorded. These observations were completed by noting any signs of fear, irritability, aggression, vocalization, convulsion, strange behavior, and measuring body weight.

Prepulse inhibition

After SHIRPA, mice performed prepulse inhibition (PPI) of the acoustic startle response (ASR) from P60-P70, using an SR-LAB system (San Diego Instruments, San Diego, CA, USA). Tone pulse parameters were controlled by a microcomputer using the software package (SR-LAB) and interface assembly that also digitized (0-4095), rectified, and recorded stabilimeter readings. The procedures for PPI were modified from a previously published protocol (Kamath et al., 2008). Animals were placed in a Plexiglas enclosure and allowed to acclimatize to their environment

with a background noise of 70 dB for 5 min before being tested during 42 discrete trials. On the first two trials, the magnitude of the startle response to a 120 dB tone of 30 ms duration was measured. These first two startle tones were presented to habituate the animals to the testing procedure and thus were omitted from the data analysis. On the subsequent 40 trials, the startle tone was either presented alone or 100 ms after presentation of prepulses of 30 ms duration with intensities ranging from 0 dB to 15 dB above background noise (i.e. 70–85 dB) that varied randomly between the trials. The prepulse-pulse trials at 70dB were presented to compare with startle response at 120 dB only and were omitted from the analysis. ASR was measured at each of the six prepulse intensities over five trials; animals were randomly presented with the startle tone alone during the other 10 trials. The interval between each trial was programmed to a variable schedule with an average duration of 15 s (range 5–25 s). A measure of ASR amplitude was derived from the mean of 100 digitized data-points collected from stimulus onset at a rate of 1 kHz. Startle response was recorded for each pulse-alone and prepulse+pulse trial. Startle magnitude was calculated as the average of the startle responses to the pulse-alone trials. The prepulse effectiveness at suppressing ASR was expressed as a percentage based on the mean amplitude of responses to ten startle tones alone relative to those recorded under the five prepulse conditions (n=5 per condition) where: %PPI= 100- (startle response for prepulse + pulse trial x 100% / startle response for pulse-alone trial).

Supporting Figure Legends

Supp. Figure 1: Effects of Poly I:C on microglial distribution and morphology in the prefrontal cortex. (A-D) Low magnification (20x) pictures showing the density of IBA1-stained microglial cells. Examples of microglial clustering are encircled in (B). (E-K) Microglial density, spacing index, clustering, morphological index, arborization area, cell body area, and cell body circularity are shown. n=5-6 mice per sex in all groups. Scale bar: 50µm.

Supp. Figure 2: Effects of Poly I:C on microglial distribution and morphology in the cerebellum. (A-D) Low magnification (20x) pictures showing the density of IBA1-stained microglial cells. (E-K) Microglial density, spacing index, clustering, morphological index, arborization area, cell body area, and cell body circularity are shown. n=5-6 mice per sex in all groups. Scale bar: 50µm.

Supp. Figure 3: Additional data pertaining to the effects of Poly I:C on microglial ultrastructure in the hippocampus DG upon E9.5 paradigm. (A-D) Microglial process perimeter, process circularity, number of vacuoles, and number of contacts with synaptic clefts. n=224-296 process profiles in 3 mice per sex in all groups. Annotations as in Figure 2.

Supp. Figure 4: Effects of Poly I:C on motor activity and anxiety-like behavior. (A-B) Open field platform and representative motion plots. (C-F) Locomotion and exploratory activities quantified. (G-I) Anxiety-like behavior was determined by measuring the time spent, number of entries into the center zone and also by counting feces after testing. n=6-8 mice per sex in all groups. $*p < 0.05$.

Supp. Figure 5: Additional data on the elevated plus maze. (A-B) Anxiety level was determined by counting number of entries and the time spent in the closed arm during testing. (C-D) Total distance traveled and immobile time of tested animals in the apparatus during testing were measured. n=6-8 mice per sex in all groups.

Supp. Figure 6: Effects of Poly I:C on novelty and spatial memory under the novel and spatial object recognition paradigms. (A) Schematic representation of the procedure used for novel object recognition (NOR) and spatial object recognition (SOR) testing. (B-C) Novelty and spatial memory were assessed by measuring the time spent interacting with new objects. n=6-8 mice per sex in all groups.

Supp. Table 1: SHIRPA primary screen in saline *versus* Poly I:C-exposed offspring

**Tests**		
	**Saline (2♂+3♀)**	**Poly I:C (2♂+3♀)**
**Viewing jar**		
Coat color	Normal: 5 (100%)	Normal: 5 (100%)
Major color	Black: 5 (100%)	Black: 5 (100%)
Minor hair color	Nothing: 5 (100%)	Nothing: 5 (100%)
Weird region	Nothing: 5 (100%)	Nothing: 5 (100%)
White belly	No: 5 (100%)	No: 5 (100%)
Hair length	Normal: 5 (100%)	Normal: 5 (100%)
Hair morphology	Normal: 5 (100%)	Normal: 5 (100%)
Respiration rate	Normal: 5 (100%)	Normal: 5 (100%)
Tremor	No: 5 (100%)	No: 5 (100%)
Body position	Standing: 5 (100%)	Standing: 5 (100%)
Activity	Slow: 1 (20%)<br>Moderate: 3 (60%)<br>Vigorous: 1 (20%)	Slow: 2 (40%)<br>Moderate: 2 (40%)<br>Vigorous: 1 (20%)
Defecation	4.00 ± 0.32	4.20 ± 0.37
Urination	No: 3 (60%)<br>Yes: 2 (40%)	No: 4 (80%)<br>Yes: 1 (20%)
**In the arena**		
Freezing response	Brief: 2 (40%)<br>Momentary: 3 (60%)	Brief: 2 (40%)<br>Momentary: 3 (60%)
Time elapsed (s)	2.40 ± 0.51	2.20 ± 0.37
No of squares	23.60 ± 1.12	25.2 ± 1.24
Piloerection	No: 5 (100%)	No: 5 (100%)
Startle response	Preyer reflex: 4 (80%)<br>Jump (1cm): 1 (20%)	Preyer reflex: 3 (60%)<br>Jump (1cm): 2 (40%)
Gait	Normal: 5 (100%)	Normal: 5 (100%)
Pelvic elevation	Normal: 5 (100%)	Normal: 5 (100%)
Tail elevation	Horizontal: 5 (100%)	Horizontal: 5 (100%)
Touch escape	Mild: 1 (20%)<br>Moderate: 4 (80%)	Moderate: 4 (80%)<br>Vigorous: 1 (20%)

**Above the arena**		
Struggling	Tail: 5 (100%)	Tail: 5 (100%)
Trunk curl	Absent: 4 (80%)<br>Present: 1 (20%)	Absent: 2 (40%)<br>Present: 3 (60%)
Limb grasping	Absent: 4 (80%)<br>Present: 1 (20%)	Absent: 2 (40%)<br>Present: 3 (60%)
**On arena and grid**		
Visual placing	Visual: 5 (100%)	Visual: 5 (100%)
Grip strength	Moderate: 3 (60%)<br>Vigorous: 2 (40%)	Moderate: 4 (80%)<br>Vigorous: 1 (20%)
Body tone	Slight: 5 (100%)	Slight: 5 (100%)
Head morphology	Normal: 5 (100%)	Normal: 5 (100%)
Pinna reflex	Active: 5 (100%)	Active: 5 (100%)
Pinna morphology	Normal: 5 (100%)	Normal: 5 (100%)
Corneal reflex	One blink: 5 (100%)	One blink: 5 (100%)
Toe pinch	Slight: 2 (40%)<br>Moderate: 3 (60%)	Slight: 1 (20%)<br>Moderate: 3 (60%)<br>Brisk: 1 (20%)
**Above arena and hold**		
Body length (cm)	9.10 ± 0.07	9.34 ± 0.12
Tail length (cm)	8.10 ± 0.05	8.12 ± 0.06
Tail morphology	Normal: 5 (100%)	Normal: 5 (100%)
Lacrimation	No: 5 (100%)	No: 5 (100%)
Palpebral closure	Eyes opened (100%)	Eyes opened (100%)
Cornea	Normal: 5 (100%)	Normal: 5 (100%)
Pupil	Normal: 5 (100%)	Normal: 5 (100%)
Eye size	Normal: 5 (100%)	Normal: 5 (100%)
Eye color	Normal: 5 (100%)	Normal: 5 (100%)
Eye shape	Normal: 5 (100%)	Normal: 5 (100%)
Whisker morphology	Normal: 5 (100%)	Normal: 5 (100%)
Tooth morphology	Normal: 5 (100%)	Normal: 5 (100%)
Provoked biting	Present: 5 (100%)<br>Absent: 0 (0%)	Present: 3 (60%)<br>Absent: 2 (40%)
Salivation	No: 5 (100%)	No: 5 (100%)
Heart rate	Normal: 5 (100%)	Normal: 5 (100%)
Abdominal tone	Slight: 5 (100%)	Slight: 5 (100%)
Skin color	Pink: 5 (100%)	Pink: 5 (100%)
Limb morphology	Normal: 5 (100%)	Normal: 5 (100%)

Limb tone	Slight: 1 (20%)<br>Moderate: 3 (60%)<br>Marked: 1 (20%)	Slight: 2 (40%)<br>Moderate: 3 (60%)<br>Marked: 0 (0%)
Wire maneuver	Active: 4 (80%)<br>Difficulty: 1 (20%)	Active: 5 (100%)<br>Difficulty: 0 (0%)
Righting reflex	Normal: 5 (100%)	Normal: 5 (100%)
Contact righting reflex	Yes: 5 (100%)	Yes: 5 (100%)
Negative geotaxis	Turn+climb:5 (100%)<br>Turn+freeze: 0 (0%)	Turn+climb: 4 (80%)<br>Turn+freeze: 1 (20%)
**Additional comments**		
Fear	Freeze: 5 (100%)	Freeze: 5 (100%)
Irritability	Yes: 5 (100%)	Yes: 5 (100%)
Aggression	Yes: 5 (100%)	Yes: 5 (100%)
Vocalization	Present: 4 (80%)<br>Absent: 1 (20%)	Present: 5 (100%)<br>Absent: 0 (0%)
Bizarre behavior	No: 5 (100%)	No: 5 (100%)
Convulsions	No: 5 (100%)	No: 5 (100%)
**Body ratio**		
Body weight (g)	21.80 ± 1.32	23.84 ± 2.00
Body mass index	0.26 ± 0.01	0.27 ± 0.02
Tail ratio	0.89 ± 0.01	0.87 ± 0.01

Supp. Table 2: Primers for *Mus musculus*

	Forward	Reverse
Ym1	cagctgggatcttcctacca	attctgcattccagcaaagg
Trem2	ctggaaccgtcaccatcact	aggctagaggtgacccacag
Cd45	gggttgttctgtgccttgtt	gatagatgctggcgatgat
Sod1	gagcattccatcattggccg	cgcaatcccaatcactccac
Sod2	ccgaggagaagtaccacgag	gcttgatagcctccagcaac
Nox2	agctatgaggtggtgatgttagtgg	cacaatatttgtaccagacagacttgag
Nox4	aaacacctctgcctgctcatt	gggaccttctgtgatcctcg
Gpx1	tgcaatcagttcggacacca	aaggtaaagagcgggtgagc
Catalase	gtgcccccaactattacccc	tcacacaggcgtttcctctc
Ptgs2	tgctggtggaaaaacctcgt	ggtgctcggcttccagtctt
Il1β	tgccaccttttgacagtgatg	aaggtccacgggaaagacac
Tnfα	atggcctccctctcatcagt	tttgctacgacgtgggctac
Il6	gtccggagaggagacttcac	ctgcaagtgcatcatcgttgt
Tgfβ1	acatgtggaactctaccagaaa	ctgccgtacaactccagtga
Cx3cr1	caagctcacgactgccttct	tgtccggttgttcatggagtt
Cx3cl1	ctcacgaatcccagtggctt	tttctccttcgggtcagcac
Gapdh	ggagaaacctgccaagtatga	ggtcctcagtgtagcccaag

Supp. Table 3: Changes in gene expression under E9.5 Poly I:C paradigm for cerebral cortex

	Male		Female	
	Saline	Poly I:C	Saline	Poly I:C
Trem2	1.00±0.10	0.90±0.17	0.98±0.07	1.14±0.14
Cd45	1.00±0.15	0.93±0.06	1.07±0.20	0.88±0.07
Ym1	1.00±0.24	0.98±0.28	0.70±0.09	0.78±0.24
Tgfβ1	1.00±0.04	0.90±0.07	0.79±0.08	0.80±0.12
Tnfα	N.D	N.D	N.D	N.D
Il6	1.00±0.13	2.03±0.60	1.26±0.22	1.12±0.26
Sod2	1.00±0.08	1.05±0.05	0.99±0.05	1.05±0.04
Catalase	1.00±0.09	0.91±0.07	0.89±0.10	0.97±0.10
Gpx1	1.00±0.11	0.91±0.01	1.06±0.13	1.06±0.20
Nox4	1.00±0.13	0.99±0.09	1.19±0.12	1.28±0.15
Cx3cr1	1.00±0.14	1.02±0.14	0.92±0.16	1.21±0.26
Cx3cl1	1.00±0.03	1.16±0.10	1.08±0.09	1.03±0.11

N.D = Non-determined. n = 4-5 animals per sex in all groups.

Supp. Table 4: Changes in gene expression under E9.5 Poly I:C paradigm for cerebellum

	Male		Female	
	Saline	Poly I:C	Saline	Poly I:C
Trem2	1.00±0.16	0.77±0.28	0.84±0.20	0.79±0.15
Cd45	1.00±0.18	0.87±0.11	0.62±0.14	0.70±0.10
Ptgs2	1.00±0.23	0.97±0.17	0.53±0.08	0.61±0.04
Tgfβ1	1.00±0.04	0.78±0.22	0.66±0.08	0.73±0.05
Tnfα	N.D	N.D	N.D	N.D
Il6	1.00±0.24	2.36±0.77	1.27±0.30	1.38±0.37
Sod1	1.00±0.10	0.93±0.17	1.03±0.17	1.01±0.09
Sod2	1.00±0.06	0.94±0.14	1.05±0.08	0.98±0.08
Catalase	1.00±0.15	0.94±0.11	0.95±0.14	0.96±0.14
Gpx1	1.00±0.15	1.00±0.11	1.10±0.19	1.04±0.14
Nox2	1.00±0.06	0.72±0.12	0.89±0.13	0.78±0.07
Nox4	1.00±0.14	1.05±0.17	0.92±0.15	0.72±0.26
Cx3cr1	1.00±0.26	0.61±0.04	0.74±0.09	0.77±0.16
Cx3cl1	1.00±0.38	0.82±0.17	0.95±0.22	0.83±0.29

N.D = Non-determined. n = 4-5 animals per sex in all groups.

Supp. Table 5: Changes in gene expression under E9.5 Poly I:C paradigm for hippocampus

	Male		Female	
	Saline	Poly I:C	Saline	Poly I:C
Cd45	1.00±0.17	0.93±0.15	1.17±0.05	1.09±0.22
Ym1	1.00±0.45	0.44±0.12	1.14±0.39	1.74±0.55
Tgfβ1	1.00±0.21	0.83±0.12	0.82±0.11	1.11±0.25
Il1β	1.00±0.36	3.66±1.95	1.34±0.54	0.85±0.19
Tnfα	N.D	N.D	N.D	N.D
Il6	1.00±0.33	1.54±0.41	1.09±0.28	2.55±1.39
Sod1	1.00±0.09	1.09±0.06	1.23±0.05	1.30±0.17
Sod2	1.00±0.09	0.98±0.12	1.04±0.14	1.39±0.32
Catalase	1.00±0.08	0.96±0.10	1.18±0.09	1.16±0.11
Gpx1	1.00±0.12	0.95±0.08	1.00±0.08	0.92±0.08
Nox2	1.00±0.18	0.86±0.15	1.11±0.17	1.35±0.31
Nox4	1.00±0.31	0.96±0.31	1.23±0.31	0.82±0.35
Cx3cr1	1.00±0.33	0.65±0.08	0.61±0.09	0.53±0.07
Cx3cl1	1.00±0.23	1.03±0.26	0.96±0.25	1.11±0.26

N.D = Non-determined. n = 4-5 animals per sex in all groups.

References

Antunes, M., and Biala, G. (2012). The novel object recognition memory: neurobiology, test procedure, and its modifications. *Cogn Process* 13, 93-110.

Goh, J.J., and Manahan-Vaughan, D. (2013). Spatial object recognition enables endogenous LTD that curtails LTP in the mouse hippocampus. *Cereb Cortex* 23, 1118-1125.

Kaidanovich-Beilin, O., Lipina, T., Vukobradovic, I., Roder, J., and Woodgett, J.R. (2011). Assessment of social interaction behaviors. *J Vis Exp*.

Kamath, A., Al-Khairi, I., Bhardwaj, S., and Srivastava, L.K. (2008). Enhanced alpha1 adrenergic sensitivity in sensorimotor gating deficits in neonatal ventral hippocampus-lesioned rats. *Int J Neuropsychopharmacol* 11, 1085-1096.

Thomas, A., Burant, A., Bui, N., Graham, D., Yuva-Paylor, L.A., and Paylor, R. (2009). Marble burying reflects a repetitive and perseverative behavior more than novelty-induced anxiety. *Psychopharmacology (Berl)* 204, 361-373.
