# Supplementary figures and images for: Prenatal Immune Challenge in Mice Leads to Partly Sex-Dependent Behavioral, Microglial, and Molecular Abnormalities Associated with Schizophrenia
Source: Front Mol Neurosci. 2018 Feb 8;11:13. doi: 10.3389/fnmol.2018.00013 (PMC5809492; doi:10.3389/fnmol.2018.00013)

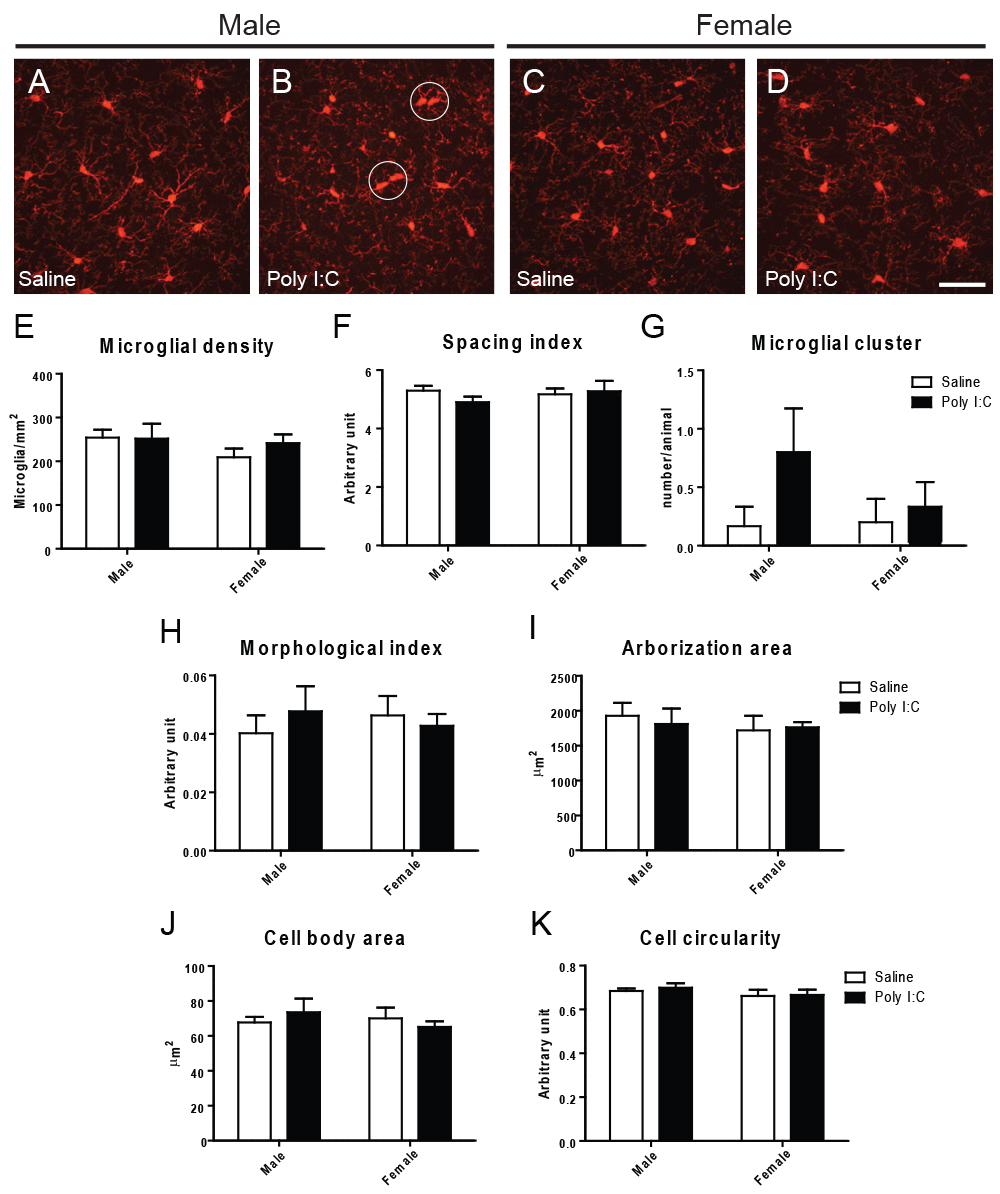

Supplement: Supplementary file 2 [file Image1.JPEG]

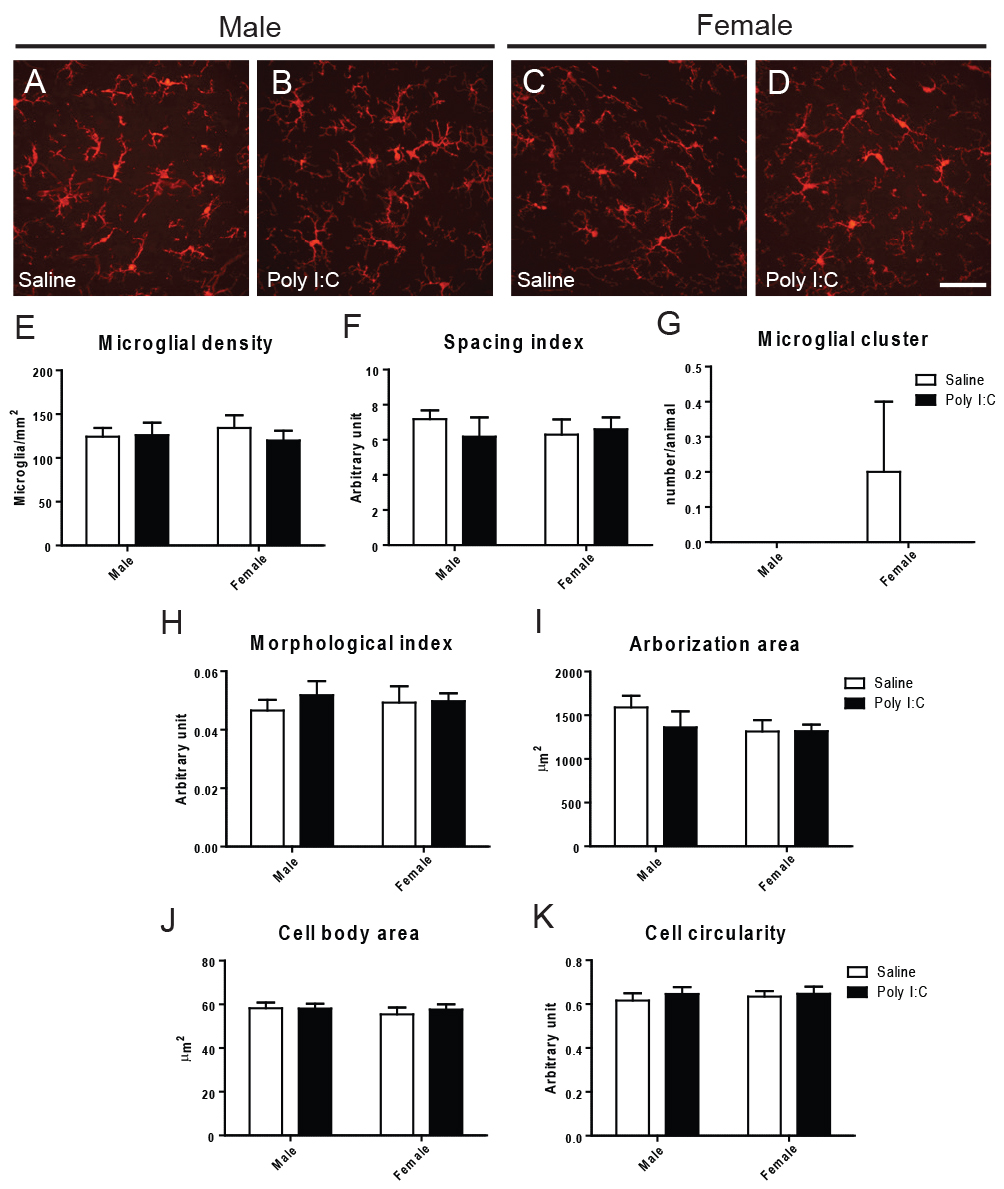

Supplement: Supplementary file 3 [file Image2.JPEG]

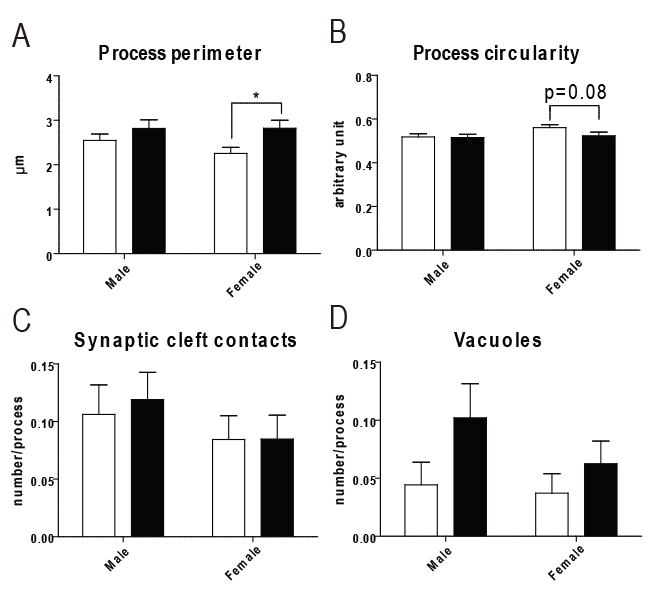

Supplement: Supplementary file 4 [file Image3.TIF]

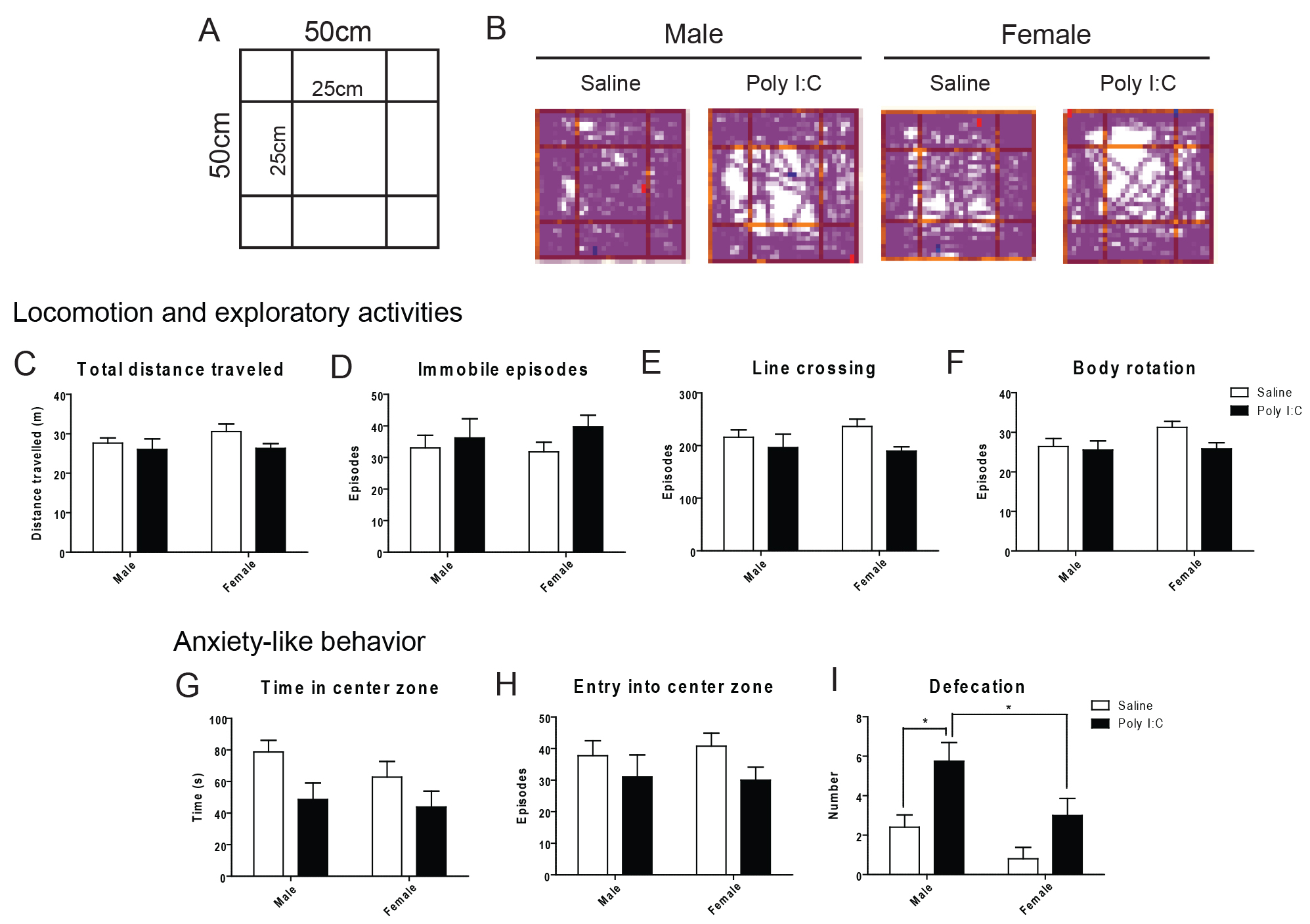

Supplement: Supplementary file 5 [file Image4.JPEG]

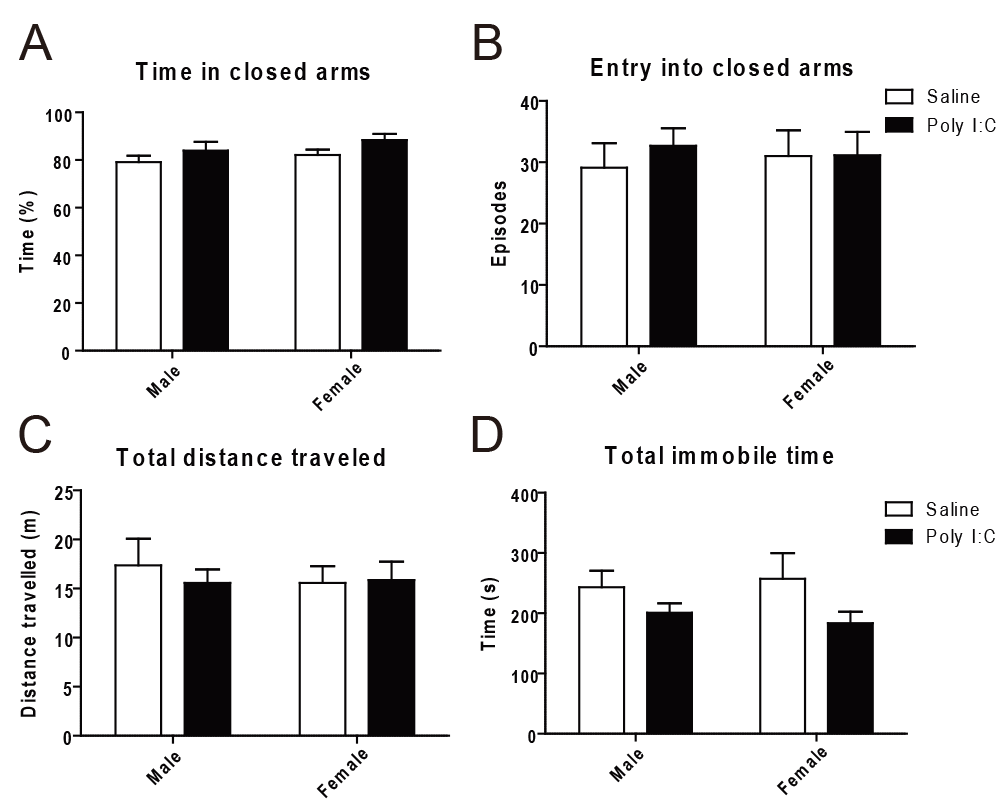

Supplement: Supplementary file 6 [file Image5.TIF]

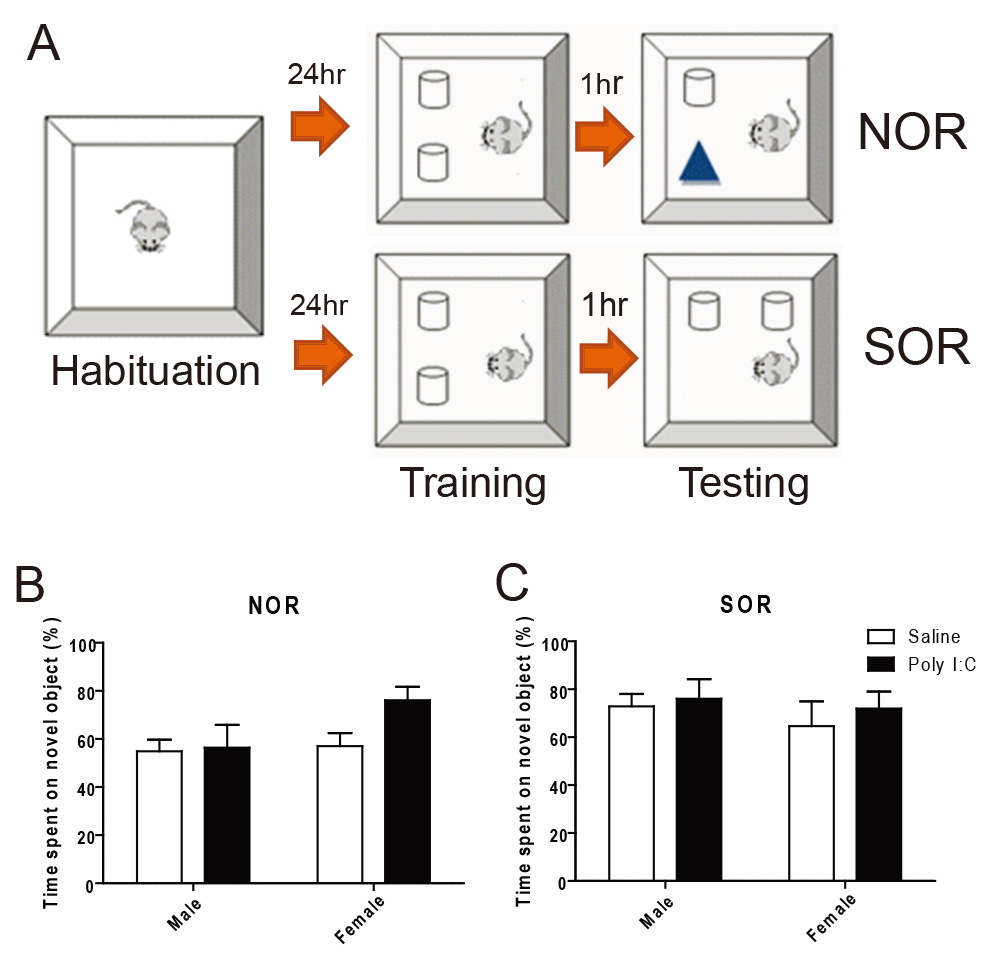

Supplement: Supplementary file 7 [file Image6.TIF]
